# Circulating Metabolites Indicate Differences in High and Low Residual Feed Intake Holstein Dairy Cows

**DOI:** 10.3390/metabo11120868

**Published:** 2021-12-14

**Authors:** Malia J. Martin, Ryan S. Pralle, Isabelle R. Bernstein, Michael J. VandeHaar, Kent A. Weigel, Zheng Zhou, Heather M. White

**Affiliations:** 1 Department of Animal and Dairy Sciences, University of Wisconsin-Madison, Madison, WI 53706, USA; mmartin37@wisc.edu (M.J.M.); praller@uwplatt.edu (R.S.P.); kent.weigel@wisc.edu (K.A.W.); 2 School of Agriculture, University of Wisconsin-Platteville, Platteville, WI 53818, USA; 3 Department of Animal Science, Michigan State University, East Lansing, MI 48824, USA; bernst84@msu.edu (I.R.B.); mikevh@msu.edu (M.J.V.)

**Keywords:** fatty acids, amino acids, oxidation, acylcarnitines, feed efficiency, untargeted metabolomics

## Abstract

Selection for more feed efficient dairy cows is key to improving sustainability and profitability of dairy production; however, underlying mechanisms contributing to individual animal feed efficiency are not fully understood. The objective of this study was to identify circulating metabolites, and pathways associated with those metabolites, that differ between efficient and inefficient Holstein dairy cows using targeted metabolite quantification and untargeted metabolomics. The top and bottom fifteen percent of cows (*n* = 28/group) with the lowest and highest residual feed intake in mid-lactation feed efficiency trials were grouped retrospectively as high-efficient (HE) and low-efficient (LE). Blood samples were collected for quantification of energy metabolites, markers of hepatic function, and acylcarnitines, in addition to a broader investigation using untargeted metabolomics. Short-chain acylcarnitines, C3-acylcarnitine, and C4-acylcarntine were lower in HE cows (*n* = 18/group). Untargeted metabolomics and multivariate analysis identified thirty-nine differential metabolites between HE and LE (*n* = 8/group), of which twenty-five were lower and fourteen were higher in HE. Pathway enrichment analysis indicated differences in tryptophan metabolism. Combined results from targeted metabolite quantification and untargeted metabolomics indicate differences in fatty acid and amino acid metabolism between HE and LE cows. These differences may indicate post-absorptive nutrient use efficiency as a contributor to individual animal variation in feed efficiency.

## 1. Introduction

Feed costs represent approximately half of the total cost of dairy production in the United States [1]. Improving feed efficiency of dairy cattle is one way to combat feed costs and improve profitability and sustainability of dairy production. Feed efficiency of dairy cattle can be measured as residual feed intake (RFI), defined as the difference between actual and predicted feed intake after accounting for known energy sinks (i.e., milk energy production, metabolic body weight, and body weight change) within a group of cows fed and managed similarly [2]. A negative RFI value indicates the cow consumed less feed than predicted and represents an efficient animal (high feed efficient, HE), whereas a positive RFI value indicates the cow consumed more feed than predicted and represents an inefficient animal (low feed efficient, LE).

Recent work indicated that RFI is a low to moderately heritable trait (h^2^ = 0.14–0.25) in Holstein dairy cattle, and that selection for RFI could contribute to genetic improvements in the feed efficiency of the Holstein breed [3,4,5,6]. Although RFI has shown utility as a genetic selection tool, the underlying mechanisms that contribute to RFI are not yet fully understood. Residual feed intake represents the unexplained variation in feed intake independent of body size, body weight gain or loss, and milk production. Previous work attributed sixty percent of the variation in RFI to six biological traits: activity, feeding behavior, uncategorized behavior, body reserve changes, rumen temperature, and digestibility [7]; however, forty percent of the variation in RFI remained unaccounted for. Preliminary work utilizing respiration chambers indicated differences in fatty acid oxidation between low and high efficient early lactation cows based on gross measures of feed efficiency [8], suggesting that a source of variation in feed efficiency could be post-absorptive nutrient metabolism. Quantifying circulating blood energy metabolites, markers of hepatic function, and acylcarnitines could provide insight into energy status and efficiency of nutrient use within the animal. Acylcarnitines are intermediates of amino acid and fatty acid metabolism and may serve as markers of incomplete oxidation [9]. The relationship between acylcarnitines and feed efficiency has not yet been explored, and it is possible it may provide insight into post-absorptive nutrient use efficiency. Additionally, further exploration using untargeted metabolomics could identify a broader range of metabolites and their associated pathways that differ between HE and LE cows.

Our objective was to identify circulating metabolites and pathways associated with those metabolites, that differ between HE and LE Holstein dairy cows using targeted metabolite quantification and untargeted metabolomics. We hypothesized that HE animals would have a circulating profile of metabolites that would be indicative of improved nutrient use efficiency compared with their LE counterparts.

## 2. Results

Cows within the HE (*n* = 28) and LE group (*n* = 28) groups were balanced by parity, and the mean RFI for the HE group (*n* = 28) was −1.99 kg/d, whereas for the LE group (*n* = 28), mean RFI was 2.02 kg/d. The least squares means for performance variables and variables included in the RFI determination by RFI group and parity are reported in Table 1. As intended by the retrospective ranking, HE cows consumed 4 kg/day less dry matter intake (DMI; *p* < 0.001) compared to LE cows, with no differences in days in milk (DIM), milk production, body weight, or body weight change (*p* ≥ 0.18). Despite tendencies (*p* = 0.08) for increased milk fat and protein concentrations to be higher in the LE group, milk fat and protein yields were similar as was milk energy output (*p* ≥ 0.64; Table 1).

### 2.1. Blood Metabolites

Table 1 reports the least squares means of circulating blood glucose, non-esterified fatty acids (NEFA), triglycerides, and β-hydroxybutyrate (BHB) for the HE and LE groups. There was no effect of parity, RFI group, or their interaction on any of these metabolites. Table 2 reports the least squares means of circulating metabolites and markers of hepatic function: blood urea nitrogen (BUN), lactate, albumin, insulin, aspartate aminotransferase (AST), and alanine aminotransferase (ALT), as well as the AST:ALT ratio and revised quantitative insulin sensitivity check index (RQUICKI, [10]). No difference in any of the aforementioned metabolites or indexes were detected between HE and LE groups (*p* ≥ 0.13). Out of the 74 acylcarnitines measured, nine were above the level of detection in the samples (Table 2). While the concentration of free carnitine was similar between groups (*p* = 0.26), C3- and C4-acylcarnitines were lower (*p* = 0.01) in HE cows compared with LE cows.

### 2.2. Untargeted Metabolomics

**Figure 1:**
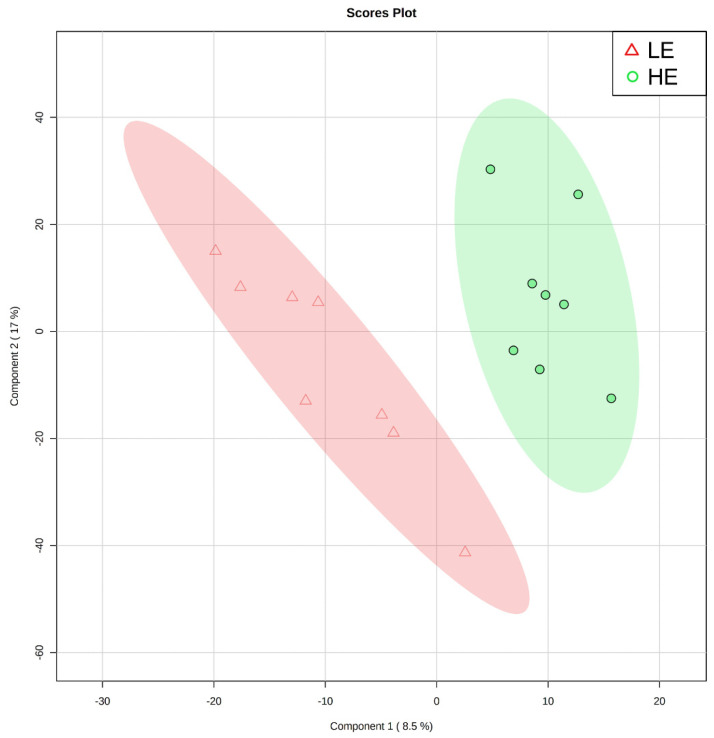
Partial least squares-discriminant analysis score plot of the first two principal components for untargeted blood plasma metabolomic analysis of low feed efficient (LE; red triangles; *n* = 8) and high feed efficient (HE; green circles; *n* = 8) mid-lactation multiparous dairy cows enrolled in a 45-day feed efficiency study.

**Figure 2:**
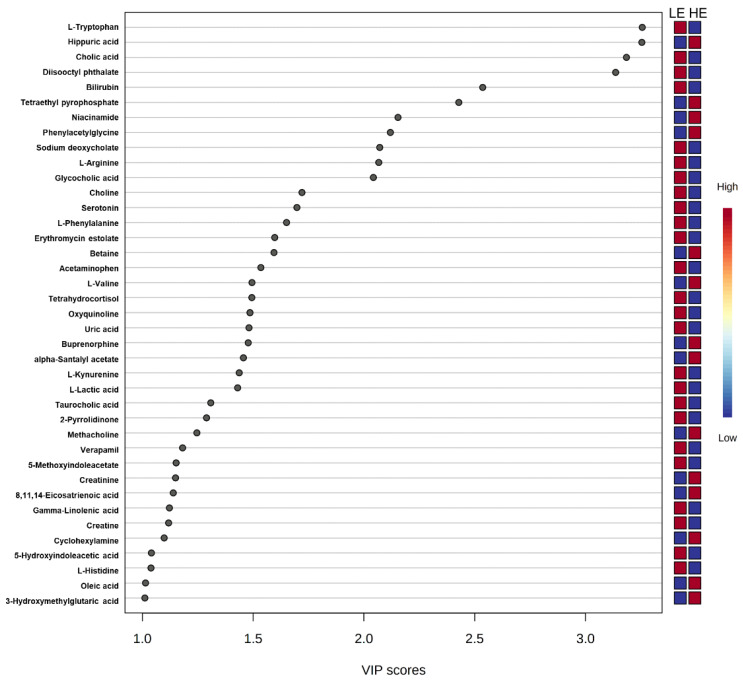
Variance importance in projection (VIP) scores from partial least squares-discriminant analysis of the 39 differential blood plasma metabolites between low feed efficient (LE; *n* = 8) and high feed efficient (HE; *n* = 8) mid-lactation multiparous dairy cows enrolled in a 45-day feed efficiency study.

**Figure 3:**
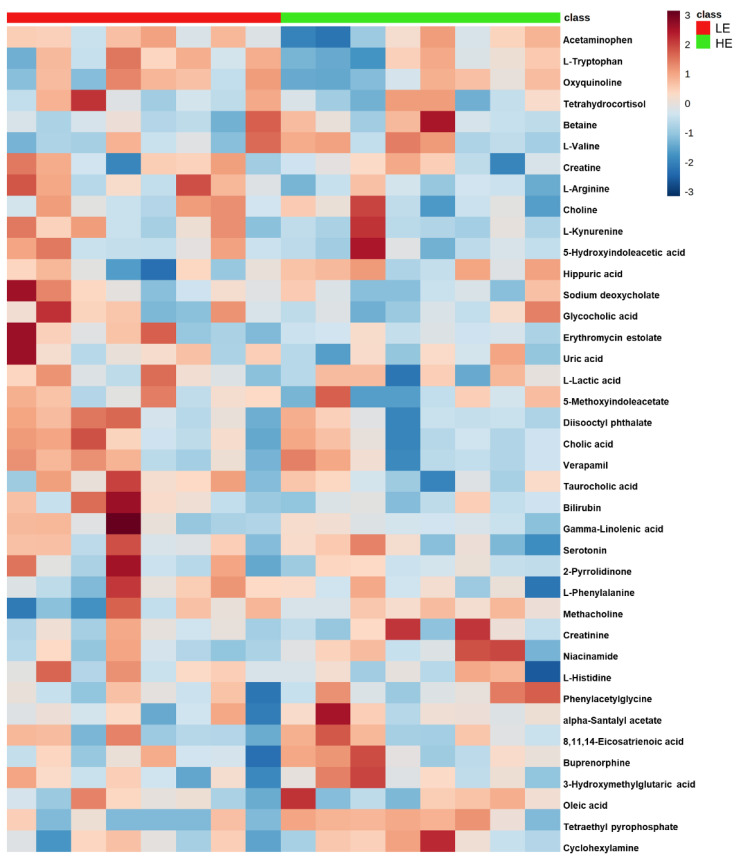
Clustered heatmap of the 39 differential blood plasma metabolites between low feed efficient (LE; red; *n* = 8) and high feed efficient (HE; green; *n* = 8) mid-lactation multiparous dairy cows enrolled in a 45-day feed efficiency study. The color scale depicts the scaled concentration of the metabolite.

**Figure 4:**
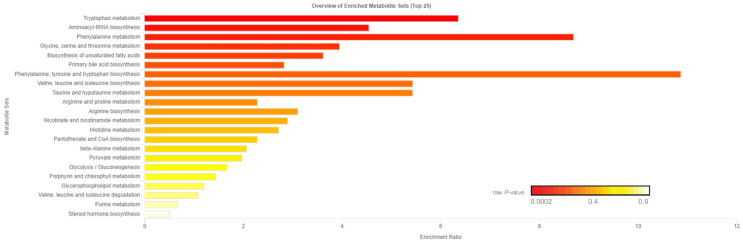
Enrichment ratio and *p*-value from enrichment analysis of pathways within the 39 differential blood plasma metabolites between low feed efficient (LE; *n* = 8) and high feed efficient (HE; *n* = 8) mid-lactation multiparous dairy cows enrolled in a 45-day feed efficiency study. Tryptophan metabolism pathway false-discovery rate corrected *p* = 0.017.

Of the 905 metabolites detected by untargeted metabolomics, 154 metabolites were uniquely identified (Table S1). As shown in Figure 1, the partial least squares-discriminant analysis (PLS-DA) score plots clearly differentiated HE and LE cows (cross-validated R^2^ = 0.99 based on 5 components), with the first five components accounting for 53.2% of the variation. Metabolites were considered differential between RFI groups when metabolite variable importance in projection (VIP) score > 1. Thirty-nine differential metabolites were identified, of which 25 were decreased and 14 were increased in HE cows compared with LE cows (Figure 2). The heatmap visualization maps discrimination in levels of differential metabolites between HE and LE cows (Figure 3). Several of the differential metabolites were amino acids, fatty acids, bile acids, and products of catabolism. Of the seven amino acids (L-tryptophan, L-arginine, L-phenylalanine, L-valine, L-kynurenine, L-histidine, and creatine), six were lower in HE cows compared with LE cows (Figure 2). Concentrations of gamma-linolenic acid (C18:3 n-6) and bile acids (cholic acid, glycocholic acid, and taurocholic acid) were lower in HE cows, whereas concentrations of oleic acid were greater in HE cows compared with LE cows (Figure 2). The differential metabolites were retained and used for pathway enrichment analysis of 22 metabolic pathways from the Kyoto Encyclopedia of Genes and Genomes (KEGG) pathway database. The tryptophan metabolism pathway had an enrichment ratio > 5 with a false-discovery rate corrected *p* = 0.017 (Figure 4 and Table S2).

## 3. Discussion

Improvements in feed efficiency of animal production over the last 100 years has primarily been through increasing production to increase dilution of maintenance [2]; however, with diminishing returns on this effort, future gains will need to be more targeted. Current progress is being made through genetic selection of more efficient animals [3,4], although the underlying mechanisms contributing to efficiency are not fully understood. Further understanding of the sources of individual animal variation in RFI would allow for more informed genetic selection decisions and could allow for optimized nutritional programs that exploit those differences. Within the current research, we strived to understand differences in circulating metabolites that would indicate tissue level metabolism contributing to differences between low feed efficient (LE) and high feed efficient (HE) cows.

Although the circulating concentration of any metabolite represents the difference between the entry into and exit from the plasma pool, the balance can be indicative of flux and provides insight into metabolic pathways that warrant investigation. In the current research, we used such concentrations to indicate which overall metabolic pathways should be further explored in future studies. Concentrations of circulating energy metabolites and activities of liver enzymes can give insight into energy status, insulin sensitivity, and liver health [10]. In the current research, no differences were detected in energy metabolites (glucose, BHB, triglycerides, or NEFA) between LE and HE cows, which is in agreement with previous research in mid-lactation cows divergent in RFI [11]. In a previous study that aimed to predict DMI and RFI in mid-lactation cows, blood glucose, BHB, triglycerides, and NEFA were quantified, but when added to predictive models of DMI and RFI, they failed to improve model performance [12]. Although differences in energy metabolites can be informative during periods of physiological change and negative energy balance, the lack of differences in mid-lactation cows in the present study was likely due to neutral or positive energy balance of the cows at this stage. Regardless, given a 4 kg/day difference in DMI in the current study, it is surprising that circulating energy metabolites that serve as key milk component precursors did not differ, given that nutrient supply is likely different between LE and HE groups. Previous research has attributed variation in RFI to dry matter digestibility [7,13], yet other studies have failed to detect differences in dry matter digestibility between LE and HE cows [11,14]. Aside from previously identified sources of variance in RFI (activity, feeding behavior, uncategorized behavior, body reserve changes, rumen temperature, and digestibility [7]), it is possible that a novel source of variation could be explained by tissue-level nutrient uptake or nutrient use efficiency. To address this, future research should investigate tissues such as the liver, muscle, and mammary gland and determine if these tissues are more efficient at extracting or utilizing nutrients in HE cows.

In the absence of tissue samples, a broader range of metabolites can provide insight into differences in metabolism between HE and LE cows. Of specific interest, short-chain acylcarnitines are intermediates of fatty acid, amino acid, and glucose metabolism, whereas long- and medium-chain acylcarnitines are derived exclusively from fatty acid metabolism [9]. In ruminants, the large amount of short-chain fatty acids absorbed from rumen fermentation may also contribute to the short-chain acylcarnitine pool in tissues [15]. Acylcarnitines can accumulate in tissue pools and efflux into circulation, and the profile and concentration of acylcarnitines have been used as indicators of energy metabolism patterns and markers of incomplete fatty acid oxidation [9]. Lower intracellular accumulations of these intermediates may indicate a greater capacity for complete oxidation via the tricarboxylic acid (TCA) cycle and, therefore, greater efficiency of extracting energy from oxidation. Incomplete oxidation of odd-chain fatty acids and amino acids generates propionyl-CoA, which accumulates within the tissue as C3-acylcarnitine and can efflux into plasma [16,17]. Lower concentrations of C3-acylcarnitine in HE cows in the current study suggest a closer alignment of propionyl-CoA generated from oxidation and flux into the TCA cycle for complete oxidation. Similarly, lower concentrations of circulating C4-acylcarnitine in HE cows reflects lower accumulation in the mitochondria of those cows, suggesting less incomplete oxidation of short-chain fatty acid and amino acids in HE cows, and may reflect greater efficiency [18,19]. Although acylcarnitine accumulation in tissue promotes its efflux into circulation, plasma concentrations of acylcarnitines do not always align perfectly with tissue concentrations, as metabolic tissues have distinct preference and efficiency for substrate oxidation [20]. Additionally, as intermediates of metabolism, lower concentrations of acylcarnitines may also be reflective of lower supply of dietary fatty acid or amino acids. Further investigation of fatty acid and amino acid metabolism in tissues (i.e., liver and muscle) is warranted to determine if these tissues differ in complete oxidation of fatty acids or amino acids between LE and HE cows, and to determine which tissue was the main source of the greater C3- and C4-acylcarnitine concentrations in LE cows observed in the current study.

While short-chain acylcarnitines indicate differences in fatty acid or amino acid oxidation between LE and HE cows, further evidence for alterations in fatty acid metabolism is supported by untargeted metabolomics. Numerically greater concentrations of C18:1-acylcarnitine in HE cows are in alignment with greater concentrations of oleic acid (C18:1) in HE cows, as determined by the PLS-DA analysis. This may result from differences in dietary supply, rumen biohydrogenation, complete oxidation, or in lipid digestion or absorption. Further, gamma-linolenic acid (C18:3 n-6) concentrations were lower in HE cows compared with LE cows, which could reflect changes in C18:2 metabolism. Interestingly, three bile acids (cholic acid, glycocholic acid, and taurocholic acid) had lower concentrations in HE cows (Figure 2). This consistent decrease of bile acids could suggest alterations in lipid digestion or absorption from the small intestine, although the methodology used was not designed to address that hypothesis specifically. Choline, which is another key nutrient involved in multiple aspects of lipid transport and metabolism, had lower concentrations in HE cows (Figure 2). In addition to being a component of milk, choline can serve as a methyl donor to regenerate methionine in the liver via betaine-homocysteine methyltransferase, and it is a key component of lipid membranes and transport moieties (i.e., phosphatidylcholine component of very low-density lipoprotein) [21]. In contrast to the decreased choline concentrations observed in HE cows, HE cows had higher concentrations of betaine compared to LE cows, which is a choline metabolite (Figure 2). This could be suggestive of increased oxidation of choline to betaine or increased choline use in HE cows. During the pre- and postpartum periods, choline is a key nutrient in support of lipid metabolism and methylation pathways [22]; however, the role of choline during the rest of lactation is less well characterized. The differences in choline and betaine observed in the current study could be suggestive of differences in supply of choline but may also be reflective of a difference in demand and use of these metabolites that may contribute to feed efficiency.

Seven amino acids, including five essential amino acids, differed between LE and HE cows (Figure 2). Other than valine, a branched chain amino acid, the other six amino acids had lower concentrations in HE cows. Differences in concentrations of amino acids between LE and HE cows may be a result of differences in supply of amino acids, ruminal microbial production, or tissue-specific preferential use of amino acids between LE and HE cows, the former of which is supported by differences in DMI. In contrast, increased concentrations of valine in HE cows, despite lower DMI, may be indicative of sparing of valine. This is supported by lower concentrations of C3- and C4-acylcarnitine noted above, which are intermediates of branched-chain amino acid (valine, leucine, isoleucine) catabolism [17]. In the pathway enrichment analysis, the tryptophan metabolism pathway was significantly different between LE and HE cows, which may also support differences in preferential use of specific amino acids by tissues. The primary pathway of tryptophan degradation is via the kynurenine pathway in the liver, where 95% of available tryptophan is degraded [23]. A decreased concentration of tryptophan and its main catabolite, kynurenine, suggest an altered flux through the kynurenine pathway of HE cows compared with LE cows.

The current work is the first to investigate the circulating profile of metabolites between HE and LE cows as it contributes to variation in feed efficiency. Combining results from the targeted metabolite quantification and untargeted metabolomics provides further insight into understanding post-absorptive nutrient metabolism and nutrient use efficiency as a source of variation in RFI. Although differences in DMI between HE and LE groups might be partially responsible for the observed differences in metabolites, these results could be suggestive of altered complete fatty acid and amino acid oxidation between HE and LE cows. Future studies investigating specific tissues that may contribute to differences in fatty acid and amino acid metabolism between HE and LE cows are warranted.

## 4. Materials and Methods

### 4.1. Animals and Diets

All animal protocols were approved by the University of Wisconsin-Madison College of Agricultural and Life Sciences Animal Care and Use Committee (IACUC #A005658). Mid-lactation Holstein dairy cows (*n* = 188, 120 multiparous, 68 primiparous) between 50 and 200 DIM were enrolled across three replicated studies of 45 d duration. Each study had the sampling schedule and protocols as detailed below. The first study was conducted from July to August of 2018 and had 63 cows, the second from September to November of 2018 with 61 cows, and the third from April to May of 2019 with 64 cows. Cows were housed in one pen of a freestall barn bedded with sand at the University of Wisconsin-Madison Emmons Blaine Dairy Cattle Research Center. All cows were fed the same total mixed ration (TMR) diet ad libitum that was formulated to meet or exceed all requirements for a 650 kg lactating cow based on the NRC (2001) [24]. The TMR was mixed once daily at 1100 h and distributed twice daily at 1100 h and 1500 h via electronic intake control bins (*n* = 32; Insentec, Marknesse, the Netherlands). Refusals were targeted at 10% and were emptied daily before morning feeding.

### 4.2. Sample Collection and Anlaysis

Samples of individual feedstuffs and TMR were collected weekly. Weekly TMR and individual feed samples were dried for 24 h in a 105 °C forced-air oven to determine dry matter. Weekly samples of individual feeds were dried at 55 °C in a forced-air oven for 48 h, ground to pass a 1 mm screen (Wiley Mill, Arthur H. Thomas, Philadelphia, PA, USA), and composited across each replicate. Individually composited feed samples were analyzed for nutrient composition at a commercial lab (Dairyland Laboratories, Inc., Arcadia, WI, USA) as described previously [12]. Diet composition and nutrient analysis are presented in Table S3. Daily intakes were recorded electronically via electronic roughage intake control bins, and DMI was calculated as the weekly dry matter of the TMR multiplied by the daily as-fed feed intake.

Cows (*n* = 188) were milked twice daily at 0400 h and 1500 h. Individual cow milk yield was recorded electronically at each milking, and milk samples were taken weekly at four consecutive p.m. and a.m. milkings. Milk samples were preserved with 2-bromo-2-nitropro-pane-1,3-diol (Advanced Instruments Inc., Norwood, MA, USA), and sent to a commercial laboratory (AgSource, Menominee, WI, USA) for analysis of milk fat, true protein, and lactose by Fourier transform infrared spectrometry using the FOSS MilkoScan FT6000 (FOSS Analytical, Eden Prairie, MN, USA). Component yields for each milking session were calculated by multiplying milk yield by the milk composition, and daily yields of components were calculated as the sum of a consecutive p.m. and a.m. milking. Energy content of the milk (MilkE, Mcal/d) was determined using the equation derived from the NRC (2001, Equations (2)–(15)): [(9.29 × milk fat [kg]) + (5.63 × true protein [kg]) + (3.95 × lactose [kg])] with the coefficient for true protein instead of crude protein.

Body weights (BW) were recorded for three consecutive days at the beginning (wk 1), middle (wk 4), and end (wk 7) of each study replicate. Metabolic body weight was calculated as BW^0.75^. Daily BW change was calculated using the LINEST function in Microsoft Excel Version 2111 (Microsoft Corp., Redmond, WA, USA) of each BW and date measured. During the same weeks as BW measurements, body condition score (BCS) was independently recorded by three trained individuals using a 5-point scale with quarter-point increments [25].

#### 4.2.1. Blood Metabolites

Blood samples were collected from the coccygeal vessel from all cows (*n* = 188) in the final weeks of each replicate, immediately after the morning milking and before feeding. Blood samples were collected in tubes containing potassium oxalate and 4% sodium fluoride (BD Vacutainer, Franklin Lakes, NJ, USA) for plasma separation and into tubes without additives (BD Vacutainer, Franklin Lakes, NJ, USA) for serum separation. Plasma was separated from whole blood by centrifugation at 2000× *g* at 4 °C for 15 min. Serum was separated from whole blood by centrifugation at 2500× *g* at 20 °C for 15 min. Plasma and serum aliquots were stored at −20 °C until analysis. Serum was used to quantify ALT and AST activities, as well as albumin concentrations. Plasma was used to quantify glucose, NEFA, triglycerides, BHB, BUN, lactate, insulin, and acylcarnitine concentrations. Concentrations of albumin (C244-01, Catachem), BUN (C264-03, Catachem), lactate (C454-01, Catachem), glucose (C124-06, Catachem), BHB (C444-OA, Catachem), and activities of AST (C164-0A, Catachem) and ALT (C164-0A, Catachem) were quantified using Catachem VETSPEC reagents on the CataChemWell-T AutoAnalyzer (Catachem, Awareness Technologies, Oxford, CT, USA), as described previously [26,27]. Concentrations of triglyceride (C116-0A, Catachem) and NEFA (C514-0A) were quantified using Catachem VETSPEC reagents in modified protocols and NEFA was quantified using a Synergy H1 Hybrid Spectrophotometer (BioTek, Winooski, VT, USA), as described previously [27]. Plasma insulin concentrations were quantified using bovine ELISA kits (Mercodia Immunoassays and Services, 10–1201–01, Uppsala, Sweden) according to the manufacturer’s protocol. Reference pool samples of each sample type were used for assay quality control. Inter-assay coefficients of variation were 7.0, 4.5, 4.9, 6.0, 4.5, 2.5, 4.2, 1.5, 1.7, and 5.1% for glucose, NEFA, triglyceride, BHB, BUN, lactate, albumin, ALT, AST, and insulin, respectively. For all assays, intra-assay coefficients of variation did not exceed 10%. The RQUICKI [10] was calculated as: RQUICKI = 1/(log[glucose mg/dL] + log [insulin µIU/mL] + log [NEFA mmol/L]) after converting insulin to international units [28].

Quantification of acylcarnitines from plasma samples (*n* = 36) was done in collaboration with the Metabolomics Lab at the University of Illinois at Urbana-Champaign. Plasma acylcarnitines were extracted according to method described previously [29] with modifications. Plasma samples (200 µL) were spiked with 0.4 nmol d3-L-Carnitine (C0) + 0.1 nmol d3-O-Acetyl-L-carnitine·HCl (C2) + 0.02 nmol d3-O-Propionyl-L-carnitine·HCl (C3) + 0.02 nmol d3-O-Butyryl-L-carnitine·HCl (C4) + 0.02 nmol d3-O-Isovaleryl-L-carnitine·HCl (C5) + 0.02 nmol d3-O-Octanoyl-L-carnitine·HCl (C8) + 0.02 nmol d3-O-Myristoyl-L-carnitine·HCl (C14) + 0.04 nmol d3-O-Palmitoyl-L-carnitine·HCl (C16) + 0.25 nmol Glutaryl-L-carnitine-(N-methyl-d3) lithium salt + 0.05 nmol d3-3-Hydroxyisovaleryl-L-carnitine. Acetonitrile (800 µL) was added to de-proteinize, and the mixture was centrifuged at 10,000× *g* for 5 min at 4 °C. The supernatant was evaporated under nitrogen at 50 °C and resuspended in 100 µL methanol:water (85:15, *v/v*) before analyses using the Q-Exactive MS system (Thermo, Bremen, Germany). Software Xcalibur 4.1.31.9 (Thermo Fisher, CA, USA) was used for data acquisition and analysis. Mass spectra was acquired under positive electrospray ionization: sheath gas flow rate, 49; aux gas flow rate: 12; sweep gas flow rate, 2; spray voltage, 3.5 kV; capillary temp, 259 °C; and Aux gas heater temp, 419 °C. The resolution was set to 70,000 with a scan range of *m/z* 50–750. An automatic gain control (AGC) target of 1E6 with a maximum injection time of 50 ms was used. In total, 35 acylcarnitines up to 22C, 19 OH-acylcarnitines, and 20 DC-acylcarnitines were measured.

#### 4.2.2. Untargeted Metabolomics

Plasma samples (*n* = 16) were extracted with Methanol: H_2_O: isopropanol (3:2:3, *v/v/v*). After the supernatant was vaporized under nitrogen, samples were resuspended in 100 uL of Methanol: H_2_O: isopropanol (3:2:3, *v/v/v*) for subsequent LC/MS analysis. The samples were analyzed by using the Q-Exactive MS system (Thermo, Bremen, Germany) in the Metabolomics Laboratory of Roy J. Carver Biotechnology Center, University of Illinois at Urbana-Champaign. Software Xcalibur 4.1.31.9 was used for data acquisition and analysis. The Dionex Ultimate 3000 series HPLC system (Thermo, Germering, Germany) used includes a degasser, an autosampler, and a binary pump. The LC separation was performed on a Phenomenex Kinetex C18 column (4.6 × 100 mm, 2.6 μm) with mobile phase A (H_2_O with 0.1% formic acid) and mobile phase B (acetonitrile with 0.1% formic acid). The flow rate was 0.25 mL/min. The linear gradient was as follows: 0–3 min, 100% A; 20–30 min, 0% A; and 30.5–36 min, 100% A. The autosampler was set at 15 °C and the column was kept at room temperature. The injection volume was 20 μL. High-resolution mass spectra were acquired under both positive (sheath gas flow rate, 46; aux gas flow rate, 11; sweep gas flow rate, 2; spray voltage, 3.5 kV; capillary temp, 253 °C; and Aux gas heater temp, 406 °C) and negative electrospray ionization (sheath gas flow rate, 46; aux gas flow rate, 11; sweep gas flow rate, 2; spray voltage, −2.5 kV; capillary temp, 253 °C; and Aux gas heater temp, 406 °C). The resolution of full scan mass spectrum was set at 70,000 with the scan range of *m/z* 70–*m/z* 1050, and the AGC target was 1E6 with the maximum injection time of 200 ms. For MS/MS scan, the mass spectrum resolution was set at 17,500. The AGC target was 5E4 with the maximum injection time of 50 ms. Loop count was 10. Isolation window was 1.0 *m/z* with NCE of 25 eV and 40 eV.

Thermo software Compound Discoverer (v.2.1 SP1, Thermo Fisher, CA, USA) was used for signal analysis, which includes peak alignment, compound detection, and compound identification. The putative identification of each metabolite was made based on an accurate mass with a detection window of ≤10 ppm. Variable importance in projection scores were obtained from multivariate analysis of plasma metabolites (PLS-DA) between HE and LE cows using MetaboAnalyst 5.0 (https://www.metaboanalyst.ca/, accessed on 27 August 2021). Metabolites with VIP > 1 were considered as differential metabolites. Pathway enrichment analysis of metabolites that differed between HE and LE cows with the KEGG pathway database were performed using MetaboAnalyst 5.0 (https://www.metaboanalyst.ca/, accessed on 27 August 2021). To visualize alterations of identified metabolites in different groups, a heatmap was generated using MetaboAnalyst 5.0.

### 4.3. Statistical Analysis

#### 4.3.1. RFI Determination

Dry matter intake for each cow was regressed as a function of major energy sinks using the PROC MIXED procedure of SAS (version 9.4, SAS Institute Inc., Cary, NC, USA). The RFI model was:

DMI_ijk_ = μ + Replicate_i_ + β1_j_ × MilkE_ijk_ (Parity_j_) + β2_j_ × MBW_ijk_ (Parity_j_) + β3_j_ × ΔBW_ijk_ (Parity_j_) + β4_j_ × DIM_ijk_ (Parity_j_) + RFI_ijk_,

where DMI_ijk_ was the observed average dry matter intake of the kth cow nested within the jth parity group (primiparous or multiparous), μ is the overall mean, and Replicate_i_ is the ith replicate. MilkE_ijk_, MBW_ijk_, ΔBW_ijk_, and DIM_ijk_ are the secreted milk energy, MBW, daily change in BW, and midpoint DIM, respectively, for the kth cow within the jth parity group and ith replicate. Regression coefficients β1_j_, β2_j_, β3_j_, and β4_j_ correspond to secreted milk energy, metabolic body weight, daily change in BW, and DIM, respectively, nested within the jth parity group. The random residual, RFI_ijk_, represents the RFI of the kth cow in the jth parity group and ith replicate.

Although parity represents a source of variance in the RFI calculation, ranking cows by RFI did not result in equal representation of parity in the 15% highest and lowest RFI. Therefore, cows were sorted by RFI within parity and the 15% highest and lowest RFI were retrospectively grouped as high RFI (LE, low feed efficient) and low RFI (HE, high feed efficient), respectively. The LE and HE groups each contained 18 multiparous and 10 primiparous cows. Plasma glucose, NEFA, triglyceride, and BHB concentrations were analyzed for all cows within the LE and HE groups. Acylcarnitines, albumin, BUN, lactate, AST, ALT, and insulin were only analyzed in multiparous cows in the LE and HE groups. A subset of the LE and HE multiparous cows (*n* = 8/group) from one replicate were considered for the untargeted metabolomics.

#### 4.3.2. Production Variables and Blood Metabolite Analysis

Data analysis was performed using the PROC GLIMMIX procedure of SAS (version 9.4, SAS Institute Inc., Cary, NC, USA). The effect of RFI group and parity (primiparous or multiparous) on production variables and plasma glucose, NEFA, triglyceride, and BHB were determined using linear mixed models with fixed effects of RFI group, parity, their interaction, and the random effect of study replicate (*n* = 56). The effect of RFI group on plasma acylcarnitines, albumin, BUN, lactate, AST, ALT, and insulin were determined using linear mixed models with the fixed effect of RFI group and the random effect of replicate (*n* = 36). Studentized residuals were assessed visually by plotting (linear predictor × studentized residuals, studentized residual quantile-quantile plot, and effect × studentized residuals plots) to determine if studentized residuals had equal variance, mean of zero, and a normal distribution. If these model assumptions were not met, data were transformed. The following variables were subjected to a Box–Cox transformation: NEFA, lactate, and C3-, C16-, C18-, C18:1-, DCC5-, and C4-OH-acylcarnitines. When model assumptions could not be met through traditional normalization methods, and unequal variance persisted, modeling heterogeneous variance was investigated. Potential heterogenous groups were determined by plotting model variables by studentized residuals. Several heterogenous groups were modeled and, in each instance, the model with the lowest Akaike’s information criteria that improved studentized residual plots was selected. Heterogeneous variance as a function of parity was identified for milk yield, milk protein yield, BCS, triglyceride, and BHB; as a function of RFI group for ALT and free carnitine; as a function of replicate for albumin, AST:ALT, and C2-acylcarnitine; and as a function of the interaction of replicate and RFI group for BUN, insulin, C4-acylcarnitine, and C5-acylcarnitine. Data are presented as the least square means and 95% confidence intervals. For transformed variables, the back-transformed means and 95% confidence interval are reported. Fixed effects with a *p* ≤ 0.05 were considered to have significant evidence for differences and effects with a 0.05 < *p* ≤ 0.10 were considered to have marginal evidence for differences.

## Figures and Tables

**Table 1:** Least squares means and 95% confidence intervals of performance variables and blood metabolites of low (high efficient, HE) and high (low efficient, LE) residual feed intake (RFI) mid-lactation primiparous and multiparous dairy cows enrolled in 45-day feed efficiency studies.

	RFI Group ^2^		Parity ^3^		*p*-Value ^4^		
Item ^1^	HE	LE	Primiparous	Multiparous	RFI Group	Parity	Interaction
Intake and body composition							
DMI, kg/day	25.1 [24.1, 26.0]	29.1 [28.2, 30.0]	24.0 [22.9, 25.0]	30.2 [29.4, 31.0]	<0.0001	<0.0001	0.35
DIM	115 [95, 134]	115 [95, 135]	111 [92, 130]	119 [97, 140]	0.97	0.27	0.40
BCS	3.4 [3.2, 3.5]	3.3 [3.2, 3.4]	3.4 [3.2, 3.6]	3.3 [3.2, 3.4]	0.39	0.08	0.85
Body weight, kg	686 [662, 710]	693 [669, 717]	636 [608, 662]	744 [724, 764]	0.68	<0.0001	0.92
Body weight change, kg/day	0.60 [0.27, 0.93]	0.57 [0.24, 0.90]	0.51 [0.18, 0.83]	0.66 [0.29, 1.03]	0.80	0.20	0.61
Milk production							
Milk yield, kg/day	44.5 [38.9, 50.0]	42.6 [37.0, 48.1]	35.9 [28.4, 43.5]	51.1 [45.9, 56.3]	0.29	<0.0001	0.81
Milk energy output, Mcal/day	30.0 [28.4, 31.5]	30.0 [28.1, 31.2]	26.0 [24.2, 27.8]	33.6 [32.3, 34.9]	0.79	<0.0001	0.96
Milk fat concentration, %	3.4 [2.6, 4.2]	3.7 [2.9, 4.7]	3.8 [3.1, 4.5]	3.3 [2.5, 4.1]	0.08	0.01	0.72
Milk fat yield, kg/day	1.48 [1.38, 1.58]	1.50 [1.41, 1.60]	1.36 [1.25, 1.47]	1.63 [1.54, 1.71]	0.72	0.0002	0.84
Milk protein concentration, %	3.05 [2.97, 3.14]	3.16 [3.07, 3.24]	3.23 [3.13, 3.32]	2.98 [2.91, 3.05]	0.08	<0.0001	0.28
Milk protein yield, kg/day	1.35 [1.15, 1.54]	1.32 [1.13, 1.52]	1.16 [0.93, 1.38]	1.51 [1.33, 1.69]	0.64	<0.0001	0.83
Blood metabolites							
Glucose, mg/dL	70.8 [62.2, 79.4]	71.3 [62.6, 79.9]	70.9 [62.6, 79.2]	71.2 [62.1, 80.2]	0.68	0.85	0.54
NEFA, mmol/L	0.14 [0.12, 0.15]	0.13 [0.12, 0.15]	0.14 [0.12, 0.15]	0.13 [0.12, 0.15]	0.47	0.79	0.12
Triglyceride, mg/dL	11.2 [10.3, 12.0]	10.8 [9.9, 11.7]	10.7 [9.8, 11.6]	11.3 [10.3, 12.2]	0.55	0.37	0.78
BHB, mmol/L	0.64 [0.58, 0.70]	0.62 [0.55, 0.68]	0.65 [0.58, 0.72]	0.60 [0.54, 0.67]	0.60	0.28	0.14

^1^ DMI = dry matter intake; DIM = day in milk; BCS = body condition score; NEFA = non-esterified fatty acids; and BHB = β-hydroxybutyrate. ^2^ HE, *n* = 28; and LE, *n* = 28. ^3^ Primiparous, *n* = 20; and Multiparous, *n* = 36. ^4^ RFI Group = effect of RFI Group; Parity = effect of parity; and Interaction = effect of the interaction of RFI Group and Parity.

**Table 2:** Least squares means and 95% confidence intervals of blood metabolites and markers of liver function for low (high efficient; HE) and high (low efficient; LE) residual feed intake (RFI) mid-lactation multiparous dairy cows enrolled in 45-day feed efficiency studies.

	RFI Group ^3^		
Item ^1^	HE	LE	*p*-Value
Energy and liver function markers			
BUN, mg/dL	18.4 [13.8, 22.9]	18.1 [13.5, 22.6]	0.72
Lactate, mg/dL	6.3 [5.3, 7.7]	7.0 [5.8, 8.8]	0.44
Albumin, g/dL	4.4 [3.9, 4.6]	4.3 [3.9, 4.7]	0.62
Insulin, µ/L	0.42 [0.33, 0.51]	0.48 [0.32, 0.63]	0.50
AST, U/L	151.5 [110.6, 192.3]	142.9 [100.6, 185.1]	0.55
ALT, U/L	29.6 [26.3, 33.0]	32.2 [30.1, 34.3]	0.18
AST:ALT	5.1 [3.2, 6.9]	4.4 [2.8, 6.0]	0.13
RQUICKI ^2^	0.52 [0.45, 0.60]	0.53 [0.45, 0.60]	0.84
Acylcarnitines, µM			
Free carnitine	5.6 [3.5, 7.6]	6.2 [4.1, 8.3]	0.26
C2-aclycarnitine	2.2 [0.5, 3.9]	2.3 [1.0, 3.7]	0.54
C3-aclycarnitine	0.9 [0.7,1.1]	1.3 [1.0, 1.6]	0.01
C4-aclycarnitine	0.24 [0.21, 0.27]	0.33 [0.28, 0.38]	0.01
C5-aclycarnitine	0.08 [0.02, 0.14]	0.07 [0.03, 0.12]	0.70
C16-aclycarnitine	0.002 [0.001, 0.005]	0.002 [0.001, 0.005]	0.86
C18-aclycarnitine	2.4 [1.2, 6.3]	2.3 [1.2, 5.9]	0.88
C18:1-aclycarnitine	0.24 [0.11, 0.75]	0.17 [0.09, 0.43]	0.16
C5-DC-acylcarnitine	1.0 [0.6, 2.6]	1.2 [0.6, 3.2]	0.47
C4-OH-acylcarnitine	0.23 [0.003, 0.80]	0.26 [0.01, 0.86]	0.11

^1^ BUN = blood urea nitrogen; AST = aspartate aminotransferase; and ALT = alanine aminotransferase. ^2^ Revised quantitative insulin sensitivity check index (Holtenius and Holtenius, 2007) calculated as: RQUICKI = 1/[log (glucose mg/dL) + log (insulin µIU/mL) + log (non-esterified fatty acids mmol/L)]. ^3^ HE, *n* = 18; and LE, *n* = 18.

## Data Availability

Feed efficiency data were collected under a specific collaborative agreement to provide data for national genetic evaluation of U.S. dairy cattle for Residual Feed Intake and Feed Saved. These data are available on request of the corresponding author and approval by the collaborators, assuming that the purpose is not to duplicate the objectives of the national collaborative project.

## Supplementary Materials

The following are available online at https://doi.org/10.5281/zenodo.5749853: Table S1, Uniquely identified metabolites from untargeted metabolomics of blood plasma between feed inefficient (LE; *n* = 8) and efficient (HE; *n* = 8) mid-lactation multiparous dairy cows enrolled in a 45-day feed efficiency study; Table S2, Differential pathways between inefficient (LE; *n* = 8) and efficient (HE; *n* = 8) multiparous dairy cows as determined by pathway enrichment analysis from the 39 differential blood plasma metabolites; and Table S3, Calculated ingredient composition and nutrient analysis of lactation diets across three replicated mid-lactation feed efficiency studies.
